# Cavernous Sinus Right Internal Carotid Artery Aneurysm With Ipsilateral Oculomotor Nerve Palsy: A Case Report

**DOI:** 10.7759/cureus.71769

**Published:** 2024-10-18

**Authors:** Jennifer M Trube, Lyudmila Sarder, Lucas Anderson, Zoya Khan, Mathew Vadaparampil

**Affiliations:** 1 Internal Medicine, Lakeland Regional Health, Lakeland, USA; 2 Medicine, Lakeland Regional Health, Lakeland, USA; 3 Critical Care, Lakeland Regional Health, Lakeland, USA

**Keywords:** cavernous anuerysm, endovascular coiling, flow diversion, internal carotid artery, oculomotor nerve (cn iii) palsy

## Abstract

Oculomotor nerve palsy, characterized by symptoms such as ptosis and restricted extraocular movements, can be a manifestation of an intracranial aneurysm. While it is commonly associated with the posterior communicating artery, it can also arise from other vascular structures, such as the internal carotid artery (ICA).

We present a 61-year-old female patient with hypertension, hyperlipidemia, and a two-year history of right-sided strabismus who presented with complaints of weakness, right-sided headache, which was ongoing for two days, as well as right-sided ptosis. Her symptoms led to the rediscovery of an ICA aneurysm within the cavernous sinus. This aneurysm was subsequently treated with flow diversion; however, it left the patient with residual ptosis and decreased extraocular movement (EOM). This case emphasizes the importance of consistent follow-up in patients with known vascular abnormalities affecting the cranial nerves.

## Introduction

Oculomotor nerve palsy refers to paralysis of the nerve controlling eye movements and is characterized by symptoms such as ptosis and restricted extraocular movements. Oculomotor nerve palsy can be a symptom of an aneurysm. Intracranial aneurysms are a feared cause of oculomotor palsy due to the risk of subarachnoid hemorrhage and subsequent death [1]. While most oculomotor nerve palsies arise due to aneurysms associated with the posterior communicating artery, these aneurysms can also arise from other vascular structures. 

Cavernous carotid artery aneurysms represent 15% of internal carotid artery aneurysms and can cause symptoms due to mass effects on the cranial nerves [2]. The cavernous sinus is clinically relevant due to the proximity of this structure to the optic nerve, the optic chiasma, and the internal carotid artery [3]. Lesions in this location are typically considered benign due to their low risk of rupture and life-threatening complications; however, symptoms including diplopia and optic neuropathy can be bothersome to patients [2]. One challenge of cavernous carotid artery aneurysm management is its risk of growth. Risk factors for growth include age and size of aneurysm at diagnosis [2]. 

The optimal timing and benefits of intervention for cavernous carotid artery aneurysms are areas in the literature that remain unclear. One reason for intervention can be bothersome and progressive symptoms, including pain and cranial nerve deficits [2]. Surgical treatment of these lesions is difficult due to their intracranial location [4]. Endovascular treatment including balloon occlusion, coiling, and more recently, flow diversion are alternative managements that show relatively good success in symptom reduction and lowering the risk of aneurysmal rupture [4].

In this case report, we present a 61-year-old female patient who presented with right-sided headache, ptosis, and restricted eye movement. Her symptoms led to the rediscovery of a previously known ICA aneurysm within the cavernous sinus that was unmonitored. This case emphasizes the importance of consistent follow-up in patients with known vascular abnormalities, particularly when they have the potential to affect cranial nerves. 

## Case presentation

A 61-year-old female with a past medical history of hypertension, hyperlipidemia, and a known history of right-sided strabismus presented with complaints of right-sided headache of two days duration, which was associated with right-sided ptosis, generalized weakness, and fatigue. Upon review of medical records, the patient's imaging was done in 2018 at an outside facility, which found a right-sided ICA aneurysm in the cavernous sinus, measuring 14 mm x 11 mm. She had been previously following this condition at another facility. They were monitoring for the occurrence of any symptoms. 

Upon arrival at the emergency department (ED), her vitals were as follows: temperature of 37 degrees Celsius, blood pressure of 164/95 mmHg, heart rate of 65 beats per minute, respiration of 18 breaths per minute, and oxygen saturation of 98% on room air. On neurological physical exam, the patient was found to have right-sided ptosis with limited extraocular movements (EOM) consistent with cranial nerve III palsy. The patient's pupils were equal and reactive to light. The patient had equal strength and sensation throughout all extremities bilaterally. 

**Figure 1:**
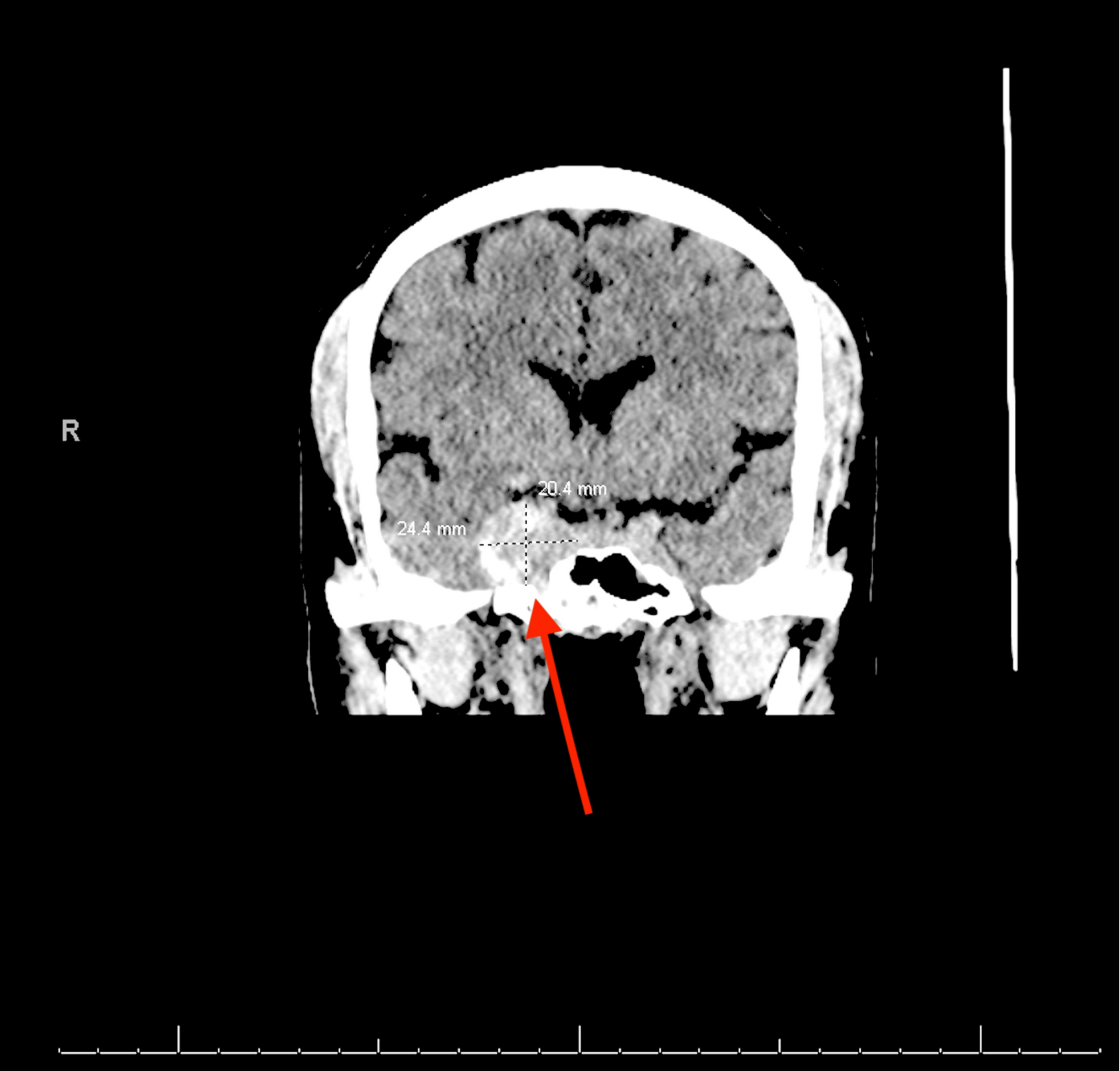
Original CT head from the emergency department The arrow points to the aneurysm within the cavernous sinus.

**Figure 2:**
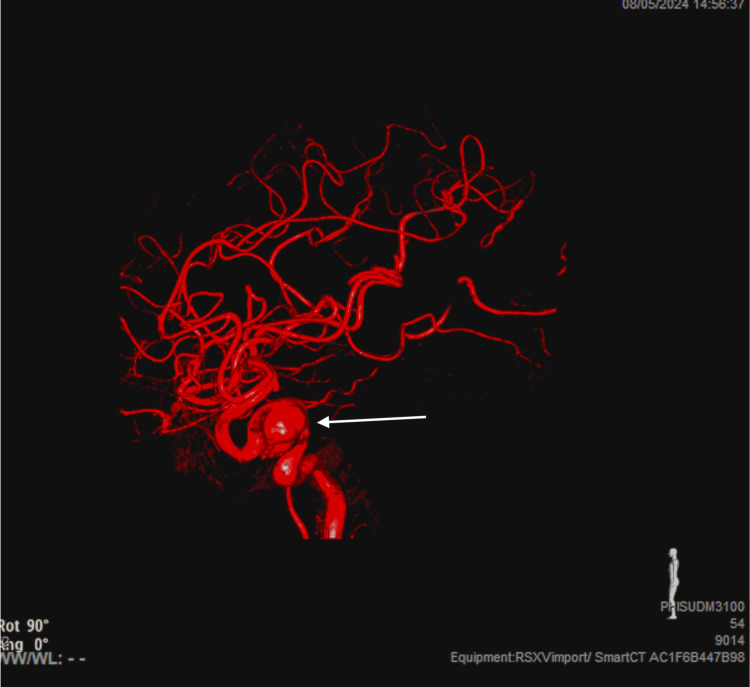
Three-dimensional (3D) pre-procedural aneurysm The arrow points to the 3D rendition of the aneurysm prior to the procedure.

The patient's lab work was unremarkable. A lumbar puncture was done to rule out a subarachnoid hemorrhage, which was negative for xanthochromia. Imaging was done in the ED revealing the right ICA aneurysm measuring 2.4 x 2.0 x 2.1 cm (Figure 1). A diagnostic cerebral angiogram was done in subsequent days demonstrating an 11 mm x 12 mm x 12 mm cavernous sinus aneurysm of the right ICA with a 7.6 mm neck and moderate tortuosity in the left cavernous segment (Figure 2). The patient was admitted to the neurological intensive care unit (ICU) for blood pressure control and continuous neurologic monitoring. Her systolic blood pressure goal was less than 140 mmHg, which was controlled with home medications and as-needed labetalol. She was given intravenous (IV) dexamethasone, oral acetaminophen every six hours, and oral tramadol for the headaches, with no significant improvement. 

**Figure 3:**
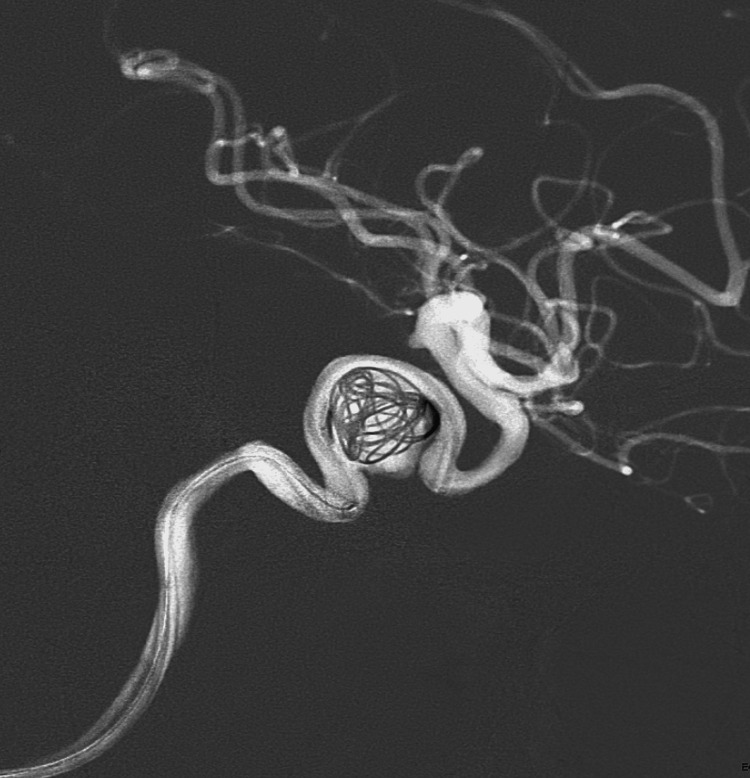
Intraprocedural flow diversion

**Figure 4:**
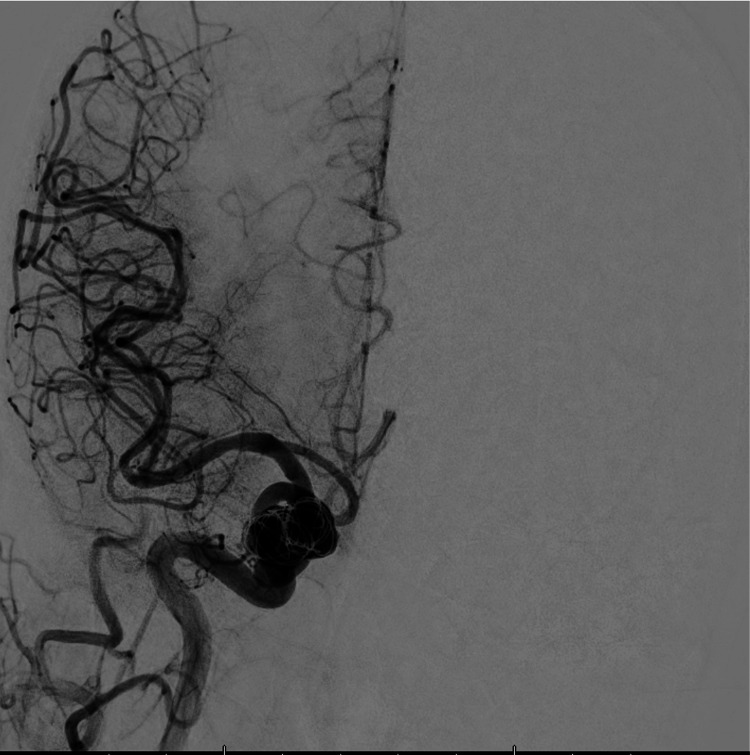
Aneurysm post-flow diversion and coiling

The patient underwent a procedure for flow diversion two days later, with coiling of the aneurysm (Figure 3). Subsequent imaging following the procedure is shown in Figure 4. Upon discharge, the patient was stable but was left with residual right-sided ptosis, decreased EOM, and headaches. The patient has a follow-up appointment scheduled with neurosurgery in the future. 

## Discussion

Oculomotor nerve palsy often arises from aneurysms in the posterior communicating artery [1]. These types of aneurysms are often considered a medical emergency due to their high risk of ruptures. Other causes of oculomotor nerve palsy include microvascular issues, trauma, compression from neoplasm, and compression from an aneurysm in the ICA. In this case, our patient presented with an oculomotor nerve palsy that originated from a right ICA aneurysm in the cavernous sinus. 

Cavernous sinus aneurysms are mostly considered benign due to their slow growth and low risk of hemorrhage [5]. However, the location of these aneurysms can often lead to impairment of ocular movement due to their proximity to the cranial nerves [5]. Similarly, our case reports a patient who presented with oculomotor cranial nerve palsy, intractable headache, and ptosis. 

The current standard of practice in the treatment of asymptomatic cavernous sinus aneurysms remains observation [6]. Many patients diagnosed with these aneurysms are elderly patients with comorbid conditions and treatment is usually diverted, even in growing lesions, until symptoms arise. Unfortunately, once symptoms manifest, patients are often left with residual effects even after treatment. Inflammation around the aneurysm can often lead to new-onset headaches or other cranial neuropathy, including diplopia, ptosis, ophthalmoplegia, and facial pain or numbness [7]. 

Treatment is often warranted when aneurysms become symptomatic or are large in size. Parent vessel sacrifice is a procedure that involves using detachable balloons or coils to occlude the entire vessel to resolve the aneurysm [8]. Carotid sacrifice achieves aneurysm occlusion over 98% of the time with an 81% reduction in diplopia but it is not without its risks [7]. There is a 5% chance of the procedure causing neurological deficits and can put stress on the existing cerebral blood flow to the brain [7]. Endovascular coiling is a reconstructive technique that involves placing a coil within the aneurysm to induce clotting within the aneurysm and prevent rupture. Flow diversion is an alternative treatment that involves placing a stent in the artery, which can reduce blood flow into the aneurysm and promote vessel wall remodeling, which can help achieve aneurysm closure and possible resolution of symptoms [7]. 

Our patient was treated with flow diversion and adjunctive coiling (Figures 2, 3). Unfortunately, at the time of discharge, our patient still suffered from right-sided ptosis and oculomotor palsy. This case indicates that treatment of these aneurysms should include follow-up imaging to ensure stability of the aneurysm.

## Conclusions

Internal carotid artery cavernous aneurysms are an important and relatively rare cause of oculomotor nerve palsy. As these aneurysms continue to grow, symptoms can result as mass-like effects, affecting quality of life in these patients. While these types of aneurysms have a low risk of rupture, consideration should be taken to monitor the growth, while serial follow up imaging and treatment should be selected for specific patients with ongoing symptoms. 
